# Response to a sexual risk reduction intervention provided in combination with hepatitis C treatment by HIV/HCV co-infected men who have sex with men: a reflexive thematic analysis

**DOI:** 10.1186/s12879-021-06003-z

**Published:** 2021-04-06

**Authors:** Patrizia Künzler-Heule, Katharina Fierz, Axel Jeremias Schmidt, Manuela Rasi, Jasmina Bogdanovic, Agnes Kocher, Sandra Engberg, Manuel Battegay, Christiana Nöstlinger, Andreas Lehner, Roger Kouyos, Patrick Schmid, Dominique Laurent Braun, Jan Fehr, Dunja Nicca

**Affiliations:** 1 Nursing Science, Department Public Health, Medical Faculty, University of Basel, Basel, Switzerland; 2 Department of Gastroenterology/Hepatology and Department of Nursing, Cantonal Hospital St. Gallen, St. Gallen, Switzerland; 3 Zurich University of Applied Sciences (ZUAS), Winterthur, Switzerland; 4 Division of Infectious Diseases, Cantonal Hospital St. Gallen, St. Gallen, Switzerland; 5 Sigma Research, London School of Hygiene and Tropical Medicine, London, UK; 6 Department of Public & Global Health, Epidemiology, Biostatistics and Prevention Institute, University of Zurich, Zurich, Switzerland; 7 School of Nursing, University of Pittsburgh, Pittsburgh, USA; 8 Division of Infectious Diseases and Hospital Epidemiology, University Hospital Basel, Basel, Switzerland; 9 Medical Faculty, University of Basel, Basel, Switzerland; 10 Institute of Tropical Medicine, Department of Public Health, Antwerp, Belgium; 11 AIDS-Hilfe Schweiz, Zurich, Switzerland; 12 Division of Infectious Diseases and Hospital Epidemiology, University Hospital Zurich, Zurich, Switzerland; 13 Institute of Medical Virology, University of Zurich, Zurich, Switzerland

**Keywords:** Hepatitis C, HIV, MSM, Sense-making, Health behavior, Qualitative

## Abstract

**Background:**

Hepatitis C virus reinfections in HIV-positive men-who-have-sex-with-men (MSM) challenge the effectiveness of antiviral treatment. To fight this problem, an adapted sexual risk reduction intervention was implemented within a hepatitis C treatment trial. Following this, the current study had two aims and describes 1) how the program was received by participants; and 2) their responses to the program regarding sexual risk taking. Based on the participants’ input, we hoped to judge the intervention’s potential for scale-up.

**Methods:**

Seventeen participants who received the sexual risk reduction intervention in addition to hepatitis C treatment were recruited for semi-structured interviews six to 12 months post-intervention. We evaluated the responses via reflexive thematic analysis and applied the concept of sense-making.

**Results:**

*Giving hepatitis C a place and living without it again* illustrates how participants received the program and how their experiences were altered by the impact of sense-making. Based on their responses, we allocated participants to three groups: 1. *Avoid risks: get rid of hepatitis C for life*. For these men, hepatitis C remained a life-threatening disease: they actively modified their risk behavior and felt supported by the intervention in maintaining their behavioral changes. 2. *Minimize risks: live as long as possible without hepatitis C.* In contrast to group 1, these men saw hepatitis C as a manageable disease. The intervention facilitated reflection on risks and how to develop behavioral changes that suited them individually. 3. *Accept risks; live with the risk of hepatitis C.* These men perceived behavioral changes as much more difficult than “easy” medical treatment. They expected to either undergo repeated rounds of treatment or stay HCV re-infected.

**Conclusion:**

These results illustrate the diversity of men’s responses and their decisions regarding sexual risk behavior after participating in a combination of antiviral treatment and a sexual risk reduction intervention. Two major aspects were identified: 1) Teachable moments, particularly at the time of diagnosis/treatment, could offer an opportunity to develop openness for behavioral change; 2) adapting sexual risk reduction interventions to sense-making patterns could help to improve its effectiveness. Support for reducing infection risk and raising awareness of preventative measures are additional benefits.

**Trial registration:**

Clinical Trial Number: NCT02785666, 30.05.2016.

## Background

Since 2014, chronic hepatitis C virus (HCV) infection has been easily curable with direct acting antivirals (DAAs), leading to enhanced survival, reduced liver-related morbidity, improved quality of life, and prevention of extrahepatic complications [1–5]. Accordingly, in 2016 the World Health Organization (WHO) set targets to eliminate HCV as a public health threat by 2030. Alongside an 80% reduction of infections, these included increasing diagnoses and treatment by 90 and 80% respectively [6].

In order to reach these targets, one key population for multiple HCV prevention strategies was identified: men who have sex with men (MSM) and who are living with HIV. Members of this group have a high anti-HCV prevalence (3–39%); and, as early as 2007, their incidence of infection was increasing by 2.34–5.11 per 100 person-years (py) [7]. Within the Swiss HIV Cohort Study (SHCS), MSM showed an 18-fold increase in HCV infections between 1998 and 2011, peaking at a rate of approximately 20 new infections per 100 py [3]. Importantly, since the introduction of direct-acting antivirals, many HIV-positive MSM have undergone successful treatment, i.e., have shown a sustained viral response after 12 weeks after the end of treatment (SVR12), and then became re-infected in a median of 100–500 days [8, 9].

To reduce HCV incidence, including reinfections, experts recommend not only scaling up HCV treatment, but also implementing interventions for risk reduction [10]. Therefore, we launched the *Swiss HCVree Trial* to test a micro-elimination strategy in this population. Micro-elimination involves precise targeting of a single sub-group’s needs [11]. In this trial, HIV-positive MSM participating in the SHCS [12] were systematically screened for HCV RNA. Positively diagnosed participants were offered treatment; those who accepted were invited to participate in a sexual risk reduction intervention [13].

Preparing the *Swiss HCVree Trial*’s, interventions for sexual risk reduction targeting MSM focused predominantly on reducing the risk of contracting or transmitting HIV via condom use. However, in the members of this subgroup of HIV-positive MSM, who are especially at risk for HCV reinfection, targeting sexual risk behavior linked closely to HCV transmission would require new and expanded approaches. Specifically, sexual practices leading to mucosal trauma, e.g., chemically prolonged receptive intercourse, receptive fisting, receptive use of sex toys, anal douching, group sex and the sharing of snorting drugs in such contexts, are strongly associated with HCV infection [14–19]. Also, increases in sex-related recreational drug use, e.g., sharing of syringes, or practices that increase the potential for anal or rectal trauma due to longer and more intense sexual encounters, often with multiple partners, has made MSM the highest-risk group for HCV infection [14, 20–23]. Over recent decades, HIV-positive MSM have increasingly engaged in condomless sexual contact with other HIV-positive MSM, leading to a higher likelihood of HCV transmission [24]. And since 2008, the concept of U=U (HIV undetectable = untransmissible) has led to ongoing decreases in condom use [25, 26]. To cover the full range of these changes, we adapted an evidence-based counseling intervention to improve self-regulation of risks associated with specific sexual behaviors and sexualized drug use. We implemented this intervention in parallel with HCV treatment [27].

Specific barriers to evaluating an intervention for sexual risk reduction were relevant to our study setting. First, the *Swiss HCVree Trial* ran in a real-world context with the goal of micro-elimination of HCV within the population of MSM with HIV/HCV co-infection. This rendered a controlled trial design impossible. Second, considering that, at the time (2016), no HCV specific intervention had been developed for this population, we systematically adapted our intervention from an HIV sexual risk reduction program [28]. This adaptation was informed by a group of four MSMs co-infected with HIV/HCV. Because of time constraints, we were unable to include a broader community in the co-creation process as described by Prinsenberg et al. [29]. Given these two limitations, we prioritized evaluating our intervention qualitatively after participants received the intervention. Our aims were to describe participants’ experiences with the program and to answer questions 1) about how the program was received by participants; and 2) their responses to the program regarding sexual risk taking. Based on participants’ input, we intended to further develop the intervention and its inherent potential for scale-up activities on this and similar initiatives.

## Method

With a constructivist orientation, we followed Braun and Clarke’s reflexive thematic analysis approach [30, 31]. Our interest was to understand the responses of MSM to this comprehensive prevention strategy, their experiences using an intervention for sexual risk reduction combined with DAAs treatment.

To aid our interpretation, we employed the concept of sense-making. Triggered by new situations, sense-making is the process of perceiving patterns within complex social environments and phenomena. These patterns can then be used to react to similar situations [32, 33]. Understanding the sense-making strategies of our participants will help us not only to understand the various participant responses to the intervention, [34] but also to identify themes, commonalities and differences between their sense-making processes [35].

### Setting and sampling

This study was embedded in the *Swiss HCVree Trial* [13, 36]. Between 2016 and 2017, 122 HIV/HCV co-infected MSM, all participants in the SHCS, accepted the offer of free DAAs treatment in one of Switzerland’s seven specialized HIV outpatient clinics. The study was conducted irrespective of DAAs restrictions existing at the time (high medication costs had led to treatment being reserved for only patients with advanced liver fibrosis or cirrhosis) [11, 37]. All MSM who reported inconsistent condom use for anal intercourse with non-steady partners during the previous year (*n* = 72) were invited to participate in the behavioral intervention [38]. At that time, this was the best-known risk behavior for HCV acquisition among SHCS participants and allowed us to more easily estimate the number of potential participants who could be included in the behavioral intervention (to allow provision of adequate resources). Fifty-one agreed to take part. As all had received both DAAs treatment and the behavioral intervention, all were eligible for this qualitative study.

We used a purposive sampling approach, which allowed us to include a diverse range of participants [39]. The final sample included MSM a) of various ages; b) across a broad range of years since their HCV and/or HIV diagnosis; c) with various numbers of HCV treatments (especially former Interferon-based treatment); d) receiving treatment at various clinics; and e) with various levels of experience with counselors. Potential participants were recruited by their responsible clinicians. Of 51 intervention participants, 21 were invited to participate in the interviews, of whom 17 agreed and provided written informed consent. In all cases of non-participation, the reason given was lack of time.

### The behavioral intervention

*HCVree and me* is a theory-based intervention using an adapted version of the information-motivation-behavioral (IMB) skills model [40, 41], social cognitive theory (SCT) [42] and theoretical aspects of cognitive neuroscience. The intervention consisted of four individual eHealth-assisted counseling sessions, which were scheduled for treatment weeks 4, 6, 8 and 12. The sessions were carried out by nurses trained in motivational interviewing techniques and the eHealth program.

The first session focused on exploring the emotions and values of participants regarding sexual behavior. This process was guided by video clips of actors portraying scenarios described by patients living with HIV/HCV. These clips were intended to evoke emotions, highlight implicit thought processes and encourage self-reflection regarding sexual risk-taking. A selection of thirteen video clips was available, from which the participant could choose up to three that were related to their personal experiences. We used these clips to both engage participants emotionally in relation to their own practices and attitudes, and present potential role models to support active learning. The second counseling session focused on perceived benefits and disadvantages of the participant’s sexual conduct. This was again supported by the video clips and interactive information (e.g., ‘What do we know about HCV risk factors?’ or ‘What does safe substance use mean?’). The third session focused on setting individual goals for behavior change. In the fourth and final session, these goals were revisited to re-assess and adapt the initial change processes.

### Data collection

Data were collected via semi-structured individual interviews 6–12 months post-intervention (end of treatment and behavioral intervention). The interviewers followed an interview guideline with open-ended questions about participants’ experiences with and perceptions of the intervention (e.g., *‘If you think back, what do you remember of the behavioral program and why?’*). We prompted participants to first describe notable situations that they experienced during the intervention, and later to reflect on any thoughts, emotions and behaviors that they perceived in relation to the program. Based on their responses, the interviewer then delved into topics such as their experience of living with HCV and/or HIV, HCV treatment, their HCV cure, and their former or current sexual risk behaviors.

Four female researchers (PKH, KF, MR, DN) conducted the individual interviews. All were academically trained nurses with experience in both qualitative data collection and developing interventions. All four researchers were also counselors in the *Swiss HCVree Trial*. Participant reflections on the intervention were able to be elicited in detail—as opposed to simple descriptions— largely due to the interviewers’ in-depth knowledge of the intervention [35]. However, to support the openness and critical feedback from participants, it was important that the interviewers had no former relationship with any of the interviewees (including as counselors).

Initial data analysis was conducted after the first three interviews, after which new questions were added based on the results from this analysis. Individual interviews lasted 37–77 min (mean: 48). According to participant preferences, eleven interviews took place at the outpatient clinic and six at their residence. All interviews were audio-recorded and transcribed verbatim.

### Data analysis

We followed Braun and Clarke’s six-phase reflexive thematic analysis approach [30, 31]. Our main analytical goal was to identify patterns of meaning across the data set that could clarify how participants responded to the program.

Analysis began with data familiarization (phase 1). Two researchers (PKH/DN) carefully read each transcript, discussed their initial notes, and then summarized their discussion in visual maps with preliminary conceptual definitions. In Phase 2, the first author began systemically identifying meaning throughout the dataset, conferring regularly with the co-authors. Similar meanings were collated into different codes. These codes helped to organize the data. Then, we developed themes based on codes, analytical memos and input from our discussions (Phase 3). Later, themes were continuously developed into meaning patterns (Phase 4). Via systematic review and comparison with our source data, we confirmed emerging meaning patterns and further identified subtle but important differences between participants (e.g., some men spoke about behavior changes before counseling started, others did not; perceptions varied regarding the severity of HCV infection). To account for such variation, we constructed groups illustrating the diversity of sense-making work in relation to the program. In Phase 5, we discussed the meaning patterns extensively within the research group. Taking place at institutional research meetings, these discussions involved one patient representative who was not a study participant. The resulting feedback led to refinement of the meaning patterns. Finally, during Phase 6, two researchers (PKH/DN) produced the study report. For transcript analysis, we used the MAXQDA Plus 2018 software (version 18.2.0).

## Results

Seventeen participants reported both on their experiences within the program and on their ongoing responses. All had achieved a sustained viral response (SVR12) with DAAs. They had a median age of 44 years (interquartile range (IQR): 41–53). They had known of their HIV infection for a median of 10.9 years (IQR 6.5–17.3) and of their HCV infection for a median of 1.6 years (IQR 1.2–4.1). Six had had experience with the earlier Interferon-based therapy prior to DAAs treatment. Two whose HCV infections were cleared by this therapy were participating in the *Swiss HCVree Trial,* receiving DAAs treatment for HCV reinfection, Table 1.

**Table 1:** Participant characteristics

Variable	Participants (***n*** = 17)
Median age (interquartile range (IQR))	44 (41–53)
White skin color, n (%)	16 (94)
Post-secondary education, n (%)	8 (47)
Number of participants living in steady partnerships, n (%)	9 (53)
Median years since HIV diagnosis (IQR)	10.9 (6.5–17.3)
Median years since HCV diagnosis (IQR)	1.6 (1.2–4.1)
Number of HCV infections, n (%)	
1	13 (76)
2	2 (12)
3	1 (6)
5	1 (6)
Former HCV treatment experience, n (%)	
Naïve	10 (59)
With Interferon	6 (35)
With DAAs	1 (6)

**Fig. 1:**
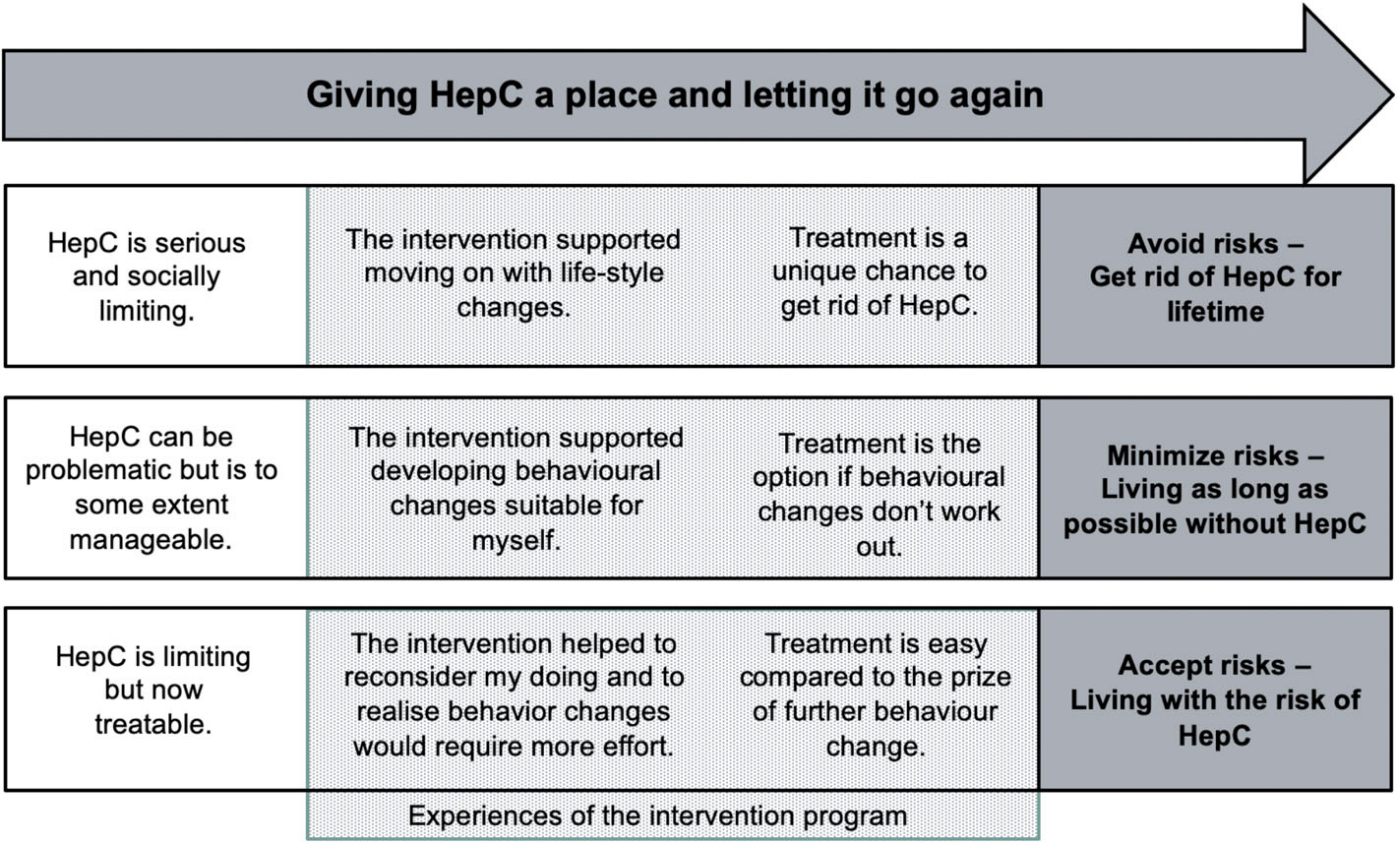
Overview of our main theme and the three subgroups to summarize the diversity of participants’ sense-making

Interviewee responses to the intervention program were influenced by their various life situations, and experiences with chronic HCV infection and its treatment, especially the current DAAs treatment within the *Swiss HCVree Trial*, influenced their responses to the intervention program. Accordingly, we identified the main theme of *Giving hepatitis C a place and living without it again* which describes how participants received the program and how their experiences were altered by the impact of sense-making. This is followed by descriptions of three explanatory subthemes, each of which accounts for a major participant group: 1) *Avoid risks: get rid of hepatitis C for life*; 2) *Minimize risks: live as long as possible without hepatitis C*; and 3) *Accept risks: live with the risk of hepatitis C* (see Fig. 1).

### Giving hepatitis C a place and living without it again

*Giving hepatitis C a place* describes how the participants approached the behavioral intervention. This was influenced by their understanding of how chronic infection would affect their lives. In addition to their experience of DAAs therapy during the study, the behavioral intervention altered their perceptions. *Living without it again* describes how participants envisioned the perceived impact of the behavioral intervention and how they felt —especially with respect to estimating the risk of possible reinfection—after being cured.

For all interviewed men, their hepatitis C diagnosis was unexpected. They reported trying to understand not only how they became infected, but also how they could evaluate the disease’s severity and its meaning for their lives. How they dealt with this phase influenced their later strategies to reduce the risk of possible reinfection. While some developed explanations that they considered valid, others reported lasting uncertainty, especially regarding transmission. One man said:

So for me, there were three relatively surprising [HCV] infections. And so therefore maybe I had more of a need than others to explore where those could have come from. Sure, there is always the risk component. But in my mind, I don’t find that part of it so large. And that’s why it’s not understandable to me. (P9, 52 years (yrs))

Later, the participants reported that, when they agreed to the behavioral intervention, they did not expect to personally benefit from it. Some had explicitly asked if they could potentially discontinue the study if they did not feel that the intervention addressed them directly (discontinuation was, of course, possible for all). However, all participants reported positive experiences in that they felt being directly and personally addressed by both the nurse counselor and the content of the program. Several participants were surprised that they could reflect seriously on themes that were relevant to them in ways that helped them to better understand their own infections and behavior. One participant expressed this experience as follows:

In many discussions, it was really about me. It was about understanding myself! And what I really want to do. [...] No one said, you should do this or that—not at all! And that was something new. (P6, 42 yrs)

While the intervention sessions were positive, the experience of being cured of hepatitis C was also tremendously important. However, this was not necessarily interpreted in a positive way: Some reported that being cured evoked a feeling of personal vulnerability from being newly infected with HCV, which they had to learn to cope with. As one man explained,

I have to deal with this whole crap again. Now, one has to be careful again. So a lot of stuff came up again. A lot of dark stuff and fears. And yeah, almost a little of the feeling that I don’t go through this again. (P16, 54 yrs)

While the main theme (*Giving hepatitis C a place and living without it again*) was central for all participants, the following paragraphs describe how the three explanatory models reflect the wide range of belief systems and sense-making, all of which were influenced by the contextual realities of participants.

#### Avoid risks: get rid of hepatitis C for life

*Avoid risks: get rid of hepatitis C for life* refers to a sense-making work shared by men who saw hepatitis C as serious and who took active steps to modify their risk behavior—even before beginning treatment and counseling in most cases. These individuals had already initiated lifestyle changes, which were felt to be supported by the intervention, and aimed to avoid risks and stay free of hepatitis C for the rest of their lives.

Men who used this sense-making work found themselves confronted with an illness that they had not even considered before their diagnosis. They reported considering HCV infection something alien—a virus relevant for intravenous drug users, not for them. The diagnosis forced them to think about hepatitis C seriously, to identify risk situations where they had been exposed, and to explain the reason for their infection. One man recalled a situation as one

where in the past you went wild with drugs, in the scene, drugs and party culture. Where night after night you took Ecstasy and went wild dancing. And then of course you had a relatively large number of sex partners, which changed up a lot. That was before [my current] relationship. (P11, 52 yrs)

They assigned these risk situations to time periods characterized by carelessness and eagerness to experiment sexually. Three gave particularly noteworthy reports of their lifestyle adjustments—of how they had to cut their risks partly because of their hepatitis C diagnoses and partly because they had formed lasting partnerships. As all but a few had received their diagnoses before any reliable therapy (DAAs) became available (median time since diagnosis: 5.8 years) most had tried to come to terms with the thought that they carried a serious communicable chronic condition.

For many, from the moment they were diagnosed*,* hepatitis C was always on their mind. One recognized it as a *“serious and socially limiting problem.”* For example, *“HepC gobbles up energy,”* leads to liver damage, and poses a huge problem for any sexual partner because of the danger of transmission.

These men embraced the possibility of being cured as wonderful. They viewed DAAs as *“a stroke of luck,” “an immense chance.”* A 45-year-old man said *“the therapy has given me a new life.”* Even after study screening and before treatment started, this group’s appreciation appeared to motivate them toward intervention-independent behavioral changes. Two men had already tried to practice sex with multiple partners only with condoms or to stop sexualized drug use because they had observed themselves becoming more reckless in recent years. Two others said they had stopped all sexual contact when their participation in the trial began.

Upon entering the intervention, based on their early reflections about the disease, transmission routes of hepatitis C and their behavioral changes, their attitude was: *“If it doesn’t help, it at least won’t make things worse.”* After all, they already had considerable knowledge and did not expect any personal gains. Many related how they had been positively surprised by the intervention, perceiving it as an environment in which they felt personally cared for and understood regarding the challenge of changing their sexual risk behavior. How one individual described it:

[The counseling] was very informative and what did it bring? You also thought about yourself again a little. That had maybe gotten a little lost lately. And it was also nice somehow to know that there are people who are at all interested. And that is for me also a nice aspect of the story. (P8, 28 yrs)

At the time of the intervention, the participants generally felt they were already moving in the right direction, but wanted to achieve and maintain *“the strict practice of safer sex.”* In the long run, they saw absolute avoidance of risk as the only way to maintain their health. This is why they used the intervention to discuss situations that were awkward and difficult for them: they wanted to be better prepared.

They also appreciated that during the intervention, according to their own personal interests, they could decide on the direction of their discussions. Among the intervention’s other benefits, they appreciated the opportunity it offered to reflect on their *“previous high-risk sex life”—*the lifestyle that had led them to acquire the disease. They recognized during the interview that their experiences with risks were something useful, an important resource in the current situation:

The light went on for me. In the sense of just thinking before you do something. Before, I didn’t have any knowledge of where you can get hepatitis C. You simply go too far and now you say to yourself: I won’t let it go so far again. (P12, 39 yrs)

A general consensus among members of this participant group was that, combined with the behavioral intervention, their successful treatment had reinforced their intention to build on and maintain the lifestyle changes. For them, using condoms for anal intercourse, while avoiding both mucosal trauma and drugs, made sense. They saw the cure of their hepatitis C as a unique chance and decided to avoid any contact with the virus in the future. Feeling relieved and happy, they experienced the cure as liberating.

One described it as *“a success that [he] was permitted to experience thanks to the therapy.”* They considered a reinfection as a personal failure, as a disgrace not only to themselves but to their doctors. One man described how the risk of reinfection was a source of fear that led to increased caution after cure:

It was strange in the beginning after the treatment. I was overly careful. I wasn’t even able to enjoy it, because I was afraid. That it turned out so well and that I don’t have it anymore. That was always a topic. (P4, 45 yrs)

Men in this group believed that their only hope for staying free of HCV was to *avoid risks.* Therefore, they had resolved not to expose themselves further. Their shared goal was never to be infected with hepatitis C again—to *get rid of hepatitis C for life.*

#### Minimize risks: live as long as possible without hepatitis C

This theme showed a sense-making process prevalent in men who considered hepatitis C a problematic but manageable disease. They described the behavioral intervention as helpful to facilitate thinking about risks and how to develop behavioral changes suitable for their long-term aim of living well without hepatitis C.

Compared with the first group, these men had only recently become aware of hepatitis C (median time since diagnosis: 1.6 years), with diagnoses received, in most cases, during regular STI testing. Unlike the earlier group, before their diagnoses, they had had vague knowledge of hepatitis C, but had paid little attention to it until they tested positive. The diagnosis had typically come as a surprise because, compared to their peers, they did not consider to be at high risk. Their diagnoses had made them uncertain of how to gauge the relative risks of various behaviors. They concluded that they must have contracted the virus in an exceptional situation. They further said that they had practiced condomless anal sex with multiple (HIV-positive) partners for years. Since the hepatitis C diagnosis was made after such a long time, they concluded that this behavior couldn’t be particularly risky and were uncertain about how to protect themselves and others:

[I regularly participated in sexual practices] without a condom. I did it like this for a long time before that, and hepatitis C didn’t happen until 2015. I surely didn’t use … [condoms] for ten years. And I had unprotected sex just as often during these ten years. (P13, 54 yrs)

Like the earlier group, these men were concerned about infecting their sex partners. However, they adhered to their original explanatory model—that they had contracted HCV during a single exceptional situation—and did not report any changes in their behavior prior to the start of the intervention program.

Similar to the first group, these men were pleased to take part in the study, and to receive the highly-effective and expensive medication free of charge. They said that when they learned of the new DAAs treatment, which was both simpler and more effective than its Interferon-based version, they concluded that *“hepatitis C can cause issues that are to some extent manageable.”*

Unlike the earlier group, these men agreed to the intervention mainly because they saw it as a possibility *“to return something because [they were] receiving DAAs with voluntary participation.”* Some also hoped to learn more about hepatitis C:

The knowledge, that’s what I was looking for. The knowledge about this, also in our community, is not really succinct or firmly understood. And for that reason, the probability of taking risks is much higher. (P3, 57 yrs)

All participants had enjoyed the behavioral intervention. In addition to the medical treatment, they appreciated the possibility of talking to a highly-knowledgeable nurse counselor who did not judge them. For this group, the knowledge gained during the intervention was a sudden insight. They were impressed by the fact that various situations could result in infection — for example, use of anal douching equipment (the act of flushing out one’s rectum with water or other liquids) or even of straws for intranasal drug consumption. From the information they received, they concluded that one of the intervention’s main messages was that *“HepC is easy to get and can also return.”* One even described the virus as particularly *“malicious.”*

Another difference between this group and the first was that the behavioral intervention motivated them to reflect on their own sexual preferences and the associated risks. During discussions with the counselor, they reflected on their personal risk situations and openly discussed possible changes to their behavior.

With their counselling sessions, they were supported in their choices for or against certain changes which could be considered by them easier to make—a dynamic reflected clearly in their perception of practicability. For example, these men did not see regular condom use as feasible, because they did not feel ready for it. Instead, members from this group chose changes they considered easily made, such as *“using gloves when fisting in a safe way* “or *“not sharing sex toys with other people.”*

Regarding behavioral changes, this group’s members also had less strict ideas than the first, adhering instead to the strategy of *“trying and choosing behavioral changes suitable for myself.”* They felt that the intervention supported them in maintaining feasibly judged behavioral changes. Some said they participated less often in sex parties, opting instead to organize non-sexual leisure weekends with friends for diversion. Others cut back on their drug consumption by carrying less money with them, or by deleting their dating apps to avoid spontaneous blind dates. One explained:

With the life I lead, it [the risk] can only be minimally reduced. And I’d rather have, for example, one encounter less and with that have the risk only once instead of twice. Rather that than … use a rubber and then not have any fun anymore. (P6, 42 yrs)

Men in this group were also tremendously impressed by the effectiveness of the new medical treatment options. However, if the behavioral changes were insufficient to prevent reinfection, they could definitely imagine another round of medical treatment as an option. Aware that they were only partially changing their risk behavior—and that this might not be enough—they changed what they believed was feasible to achieve success. One participant explained:

What I want or should do, I am absolutely still aware. I knew it before, but the program has created more awareness. But I am not so good at implementation, or actually not good at all so to speak. But I do think, I do some of the things, but just not all that I have wanted to do. (P10, 44 yrs)

For this group, risk reduction contributed importantly to living as long as possible without hepatitis C. Having chosen to *minimize risks (to the best of their perceived ability)* they knew this strategy left them vulnerable to reinfection. Compared with the first group, they made few compromises, but hoped to *live as long as possible without hepatitis C*.

#### Accept risks: live with the risk of hepatitis C

The third sense-making group included men who were highly concerned with hepatitis C more for fear of sexual rejection than health problems. They described the intervention as useful to help them reconsider their own sexual risk behavior and to realize that further behavior changes would require considerable effort to avoid reinfection—in contrast to medical treatment, which they perceived as “easy.” In this sense, they expected to either undergo repeated rounds of treatment or, if necessary, stay HCV re-infected.

As in the second group, men with this sense-making style had only known of their hepatitis C infection for a relatively short time (median 1.5 years); however, as in the first group, the diagnosis had elicited an intervention-independent, active and intensive search for information to explain and understand the infection. Two stated, for example, that they had already undergone at least one successful Interferon-based therapy and that they had then sought information to allow them to consciously protect themselves against reinfection. Based on the extent of their knowledge at that time and how they viewed the first (successfully treated) infection, they had decided on certain behavioral changes, such as “*avoiding fisting.”*

Two men living together as partners reported other experiences. As both had HIV/HCV co-infection, they saw no need to change their behavior. They described their joint status even as a relief. They could set the topic of hepatitis C aside:

What was easy for us was that we had the same thing. He was positive [HIV and HCV] and me, too. That’s why we got together, because we supported each other. Because how do you want to find a life partner that doesn’t have it, that doesn’t understand the problems? We complemented each other well. We each respected each other, supported, showed affection to one another. It was probably just as hard for him to find a life partner as it was for me—someone that accepts and takes you as you are. (P15, 34 yrs)

Among members of this group, attitudes toward the behavioral intervention reflected their intense personal search for hepatitis-C-related information prior to the study. Similar to the first group, they had low expectations of the behavioral intervention and mainly participated to please their physicians, who they believed truly worried about them. However, having already gained considerable knowledge and practiced changing some of their behavior, they did not see themselves as the right people for an intervention. Unlike the first group, but analogous to the second, they had not tried to completely eliminate the risk of reinfection but had selected easy risk-reduction adaptations. Still, two members of this group who contracted HCV again despite such changes were dumbfounded. As one said:

When you can’t pin it [the infection] down—you know, I mean—the first time it was so nice, because I knew exactly where it [hepatitis C] came from, where I got it, from whom. I knew out of which situation it came. Then it’s easy to say, ‘Ok, I’ll change something.’ But when later I stand there and the liver values are high and I can’t link it to any specific situation, then it’s difficult to change anything. (P14, 46 yrs)

These men did not participate in the intervention primarily to learn and expand their knowledge, but rather as a place where they could openly talk about past difficult situations and about their failures. Therefore, they talked with the counselor, for example, about the difficulty of disclosing their HCV status. While they acknowledged that this was an important preventive measure, they found it difficult because of the rejection they experienced as a result:

It’s hard to change much. Because in the moment you don’t want to talk about it. Because that’s when you want to party, have sex, you want to enjoy and you don’t want to say, ‘Hey stop! Hepatitis C!’ Then everything would be over. (P16, 54 yrs)

The men in this third group saw little possibility of protecting themselves more effectively in the future: similar to those in the second group, they considered strict use of condoms, monogamy or even total rejection of sexualized drug use as effective protective measures, but considered such adaptations too extreme and difficult. This was described particularly succinctly by one participant—a self-professed *“sex and drug addict”*—who acknowledged that, while the intervention made sense, it was not intensive enough for his needs.

This group saw only one feasible restriction: *“having fewer sexual encounters.”* Unlike the second group, these men knew how easily they could be infected with HCV, but insisted on continuing risky “non-negotiable behaviors” and expected to be re-infected at any time. One man called this approach—taking risks to enjoy sex—*“Russian roulette.”* This group considered the new DAAs a good and important option compared to the challenges of behavioral change. One man exemplified this attitude:

That would probably also go in that direction with hepatitis C. … It will become less expensive to treat and then it will become even less a topic for some people, like myself, to think about having sex with a condom. (P5, 54 yrs)

They recognized the great benefit of successful therapy*: “the liver gets a break.”* At the same time, though, the cure appeared to elicit ambivalence. While it greatly decreased their potential liver-related morbidity, it also meant *“having to watch out again.”* One described it anxiously as *“feeling put back to the time with HIV before the [2008] Swiss Statement”*. At that time, when condom use was strongly promoted, he experienced his sexual life as more limited and less pleasurable.

Thanks to the availability of curative therapy, members of this group hoped that all MSM would regularly be tested for HCV and receive treatment as necessary. They were convinced that this would reduce the danger of HCV infection for their sexual partners. Having rejected major behavioral changes, they intended to *live with the risk of hepatitis C,* i.e., they believed that their only reasonable course of action was to a*ccept the risks.*

## Discussion

This study adds considerably to the understanding of how HIV/HCV co- and/or re-infected men responded to one of the first HCV-specific sexual risk reduction interventions to be implemented in combination with DAAs treatment. Results show the processes engaged by participants in how they position themselves in relation to the program, as well as their sense-making regarding the intervention thereafter. We identified three sense-making groups that helped to summarize the variety of responses regarding individual sexual risk reduction appraisal, decision-making, strategies to avoid re-infection and challenges to behavioral change.

The main theme, *Giving hepatitis C a place and living without it again,* covers the continuum of sense-making, with the lasting effects from the intervention program influenced by two specific factors: the time of hepatitis C diagnosis and the effectiveness of the program—including counseling and pharmaceutical treatment—regarding the prospect of curing their HCV.

We noted that the first experience of hepatitis C diagnosis was usually unexpected and often a shock. Diagnosis led first to reflection, then to individual explanatory patterns regarding transmission and the perceived consequences of particular sexual practices. In line with previous studies [43, 44], the behavioral change resulting from such reflection varied between participants. Across the cited studies, including ours, some MSM reacted to their diagnosis by taking a sexual break or reducing sexual risk behavior; others showed little or no behavior change.

The second important experience inherent in the main pattern was the prospect of being cured of HCV. Interestingly, and in contrast to other studies’ findings, the prospect of cure also induced negative feelings in participants for various reasons. Whereas some men described feelings of shame (in relation to their physician) if a reinfection were to occur, others expressed ambivalence about once more taking the responsibility to not get infected with hepatitis C again. To our knowledge, this is the first time that negative feelings towards an HCV cure have been noted in the perceptions of MSM. This contrasts sharply with the results of a study during the era of interferon-based therapy, in which all interviewed HIV-positive MSM spoke completely positively about their HCV-free status [45]. Understanding more will require further research on this topic and the potential consequences to meet the care needs of MSM. In addition, our results emphasize the strong possibility that reinfection will lead to stigmatization, similarly described by Richmond et al. [46]. In the clinical setting, professional teams should reflect upon and discuss this potential stigma with patients.

Alongside our main theme, based on the diverse results of the participants’ sense-making work, we divided them into three broad groups: *1) Avoid risks: get rid of hepatitis C for life; 2) Minimize risks: live as long as possible without hepatitis C; and 3) Accept risks: live with the risk of hepatitis C* (see Fig. 1).

One meaningful difference was observed in the participant’s perceived susceptibility to HCV reinfection in relation to their experience with DAAs. All individuals perceived DAAs as a low-burden treatment; however, whereas two groups explained that they expected a change in outcome (from serious HCV infection to treatable), one did not. This finding might explain certain differences in the likelihood of behavior changes [42]. For example, men who had previously perceived HCV infection as a major health problem, and who therefore chose the risk avoidance sense-making strategy, were most committed to behavioral change. Those with the sense-making approach either to *Minimize* or to *Accept risks* were particularly attracted by DAAs treatment, the ease of which they weighed against the perceived difficulty of behavior change, leaving them more open to re-treatment than to behavior change. In other words, while the prospect of not having to take HCV medications again motivated some towards changing their behavior, that was not universal. This supports the findings of a recent study in which Lambers et al. [45] described the impact of perceived treatment burden on motivation towards behavior change. However, the variety of experience, ranging from *treatment instead of behavioral change,* towards *treatment as an option if risk reduction fails* to *treatment as the one single change to get rid of HCV*, has not been described so far and is informative for prevention initiatives combining treatment and counseling.

Another meaningful difference between these groups was in the risk perception of participants, namely what they experienced as their personal risks regarding re-infection. For example, men from the *Avoid risks* group seemed convinced that condomless anal intercourse or past sexual drug use had led to their HCV infection. As a consequence, they intended to protect themselves by avoiding virtually any risky situations. In contrast, many from the other two groups were convinced that it was not condomless anal intercourse that led to their HCV infection but other, more complex behaviors, e.g., using drugs, sharing sex toys or fisting without gloves. Therefore, they perceived that only changing such high-risk behaviors would offer adequate protection.

The risk minimizers, who first recognized the risks entailed by many behaviors during the sexual risk reduction intervention, perceived the elimination of those practices as feasible. In contrast, as the risk accepters had already tried such changes without success, they saw no net value in renewing their earlier attempts. Importantly, this finding is consistent with Bandura’s concept of self-efficacy [42], i.e., the principle that a person’s perceived capability to perform a target behavior influences their decisions for or against changes that depend on that behavior. This implies that intervention programs need to include components that strengthen self-efficacy, according to the groups, the dosage might be tailored.

The groups were further differentiated regarding the timing and delivery of behavior changes after study recruitment. For this intervention, we recruited only men who reported condomless anal intercourse with non-steady partners in the last year. Whereas the risk avoiders had already initiated behavior change by the start of the behavioral intervention, the other two had not. According to the Transtheoretical model of change (TTM) [47], which describes five stages of change (ranging from precontemplation to maintaining behavior changes), these men had already reached the “acting” phase at the start of the counseling intervention. They used the behavioral intervention to maintain and stabilize the changes that they had already made.

In contrast, the risk minimizers responded to their counseling with their first serious reflection on their risk behavior. This led them to target and implement behavioral changes as encouraged via motivational interviewing techniques [48]. In relation to the TTM [47], by the end of the intervention program, these men had reached the phase of either “preparation” or of “action.” Members of the *Accept risks* group described no motivation for a new attempt at behavioral change but reported reflection of their behavior during the intervention. These men either relied completely on access to successful therapy or resigned themselves to life with chronic HCV infection. Accordingly, they seemed to be trapped between the TTM phases of “precontemplation” and “contemplation.”

The diversity of responses to each step of the program illustrates that, in research studies, as in clinical practice, screening, diagnosis and treatment all offer teachable moments. That is, participants’ openness to information and reflection can arise at any point of contact; and when it does, it can serve as a fulcrum for motivational support by clinicians [49, 50]. Additionally, the sense-making attitudes of the three groups emphasize the need for related interventions to support tailoring regarding content, duration and timing. For example, by shortening the behavioral intervention for the *Avoid risks* group, we could focus more on maintaining behavioral change; and for those reporting sexualized drug use behavior, extending the behavioral intervention would allow a sharper focus on overcoming their ambivalence and initiating change towards lower-risk practices.

Considering the strengths and weakness of the methods described above, although the participant interviews were conducted 6 to 12 months post-intervention, the stories from the interviewees reflected rich, meaningful, well-remembered experiences regarding the intervention. One clear limitation was our decision to use a purposive sampling strategy instead of interviewing all 51 participants. Whereas the used strategy worked well to include individual characteristics (e.g., age distribution, years since HIV or HCV diagnosis), we did not reach maximum variation between centers, as we were unable to recruit participants from Switzerland’s French-speaking region. Therefore, our results fail to represent one major region. However, the diversity of responses to the complex intervention program (behavioral counseling and DAAs therapy) should be sufficient to support not only a subsequent mixed-methods quantitative outcome evaluation, but also the advancement and tailoring of an intervention program focusing on HCV micro-elimination.

In summary, this study’s findings indicate a need for further development regarding related interventions and clinical practice. It is essential to bear in mind that, for this subgroup of MSM co-infected with HIV and HCV, both the sexual risk reduction intervention and curative DAAs treatment influence future behavioral changes.

## Conclusion

Via an inductive interpretative approach to explore responses to a comprehensive HCV prevention initiative, this study helped us both to understand the diversity of participant responses and their decisions regarding sexual risk behavior. Participants responded to all aspects of the study, including HCV screening, diagnosis, treatment and counseling, with reflection toward behavioral change. The variety of experiences also impacted participants. Our results provide important insights into the wide range of responses after receiving a combined prevention intervention including treatment and counseling.

The results will facilitate ongoing development of this and similar programs’ behavioral interventions, particularly by identifying intervention components that can be tailored to fit each target group’s attitudes/beliefs. Impact can be tailored by adjusting how much of the intervention is received and/or how long the intervention lasts. These results also imply important recommendations for clinical practice to enhance the effectiveness of infection prevention components. Participants appreciated individual counseling; and clinicians were well-positioned to first initiate and stimulate risk-related discussions, and then in turn those discussions into teachable moments by addressing and planning concrete behavioral changes.

## Data Availability

The individual level datasets generated and/or analysed during the current study are not publicly available because open access to all SHCS data is currently not possible. This data is too dense and comprehensive to preserve patient privacy in patients with HIV infection. Free access to the data would currently not be compatible with the SHCS informed consent and with preserving patient privacy. Investigators with a request for selected data should send a proposal to the corresponding author. The provision of data will be considered by the study team and the Scientific Board of the SHCS.

## Abbreviations

DAAs: Direct acting antivirals
HCV: Hepatitis C virus
IQR: Interquartile range
IMB: Information-Motivation-Behavioral skills model
MSM: Men who have sex with men
PY: Person-years
SCT: Social Cognitive Theory
SHCS: Swiss HIV Cohort Study
SVR: Sustained Viral Response
TTM: Transtheoretical Model
Yrs: Years
